# Low Photosensitizer Dose and Early Radiotherapy Enhance Antitumor Immune Response of Photodynamic Therapy-Based Dendritic Cell Vaccination

**DOI:** 10.3389/fonc.2019.00811

**Published:** 2019-08-27

**Authors:** Bastien Doix, Natalia Trempolec, Olivier Riant, Olivier Feron

**Affiliations:** ^1^Pole of Pharmacology and Therapeutics (FATH), Institut de Recherche Expérimentale et Clinique (IREC), UCLouvain, Brussels, Belgium; ^2^Institute of Condensed Matter and Nanosciences, Molecular Chemistry, Materials and Catalysis (IMCN/MOST), UCLouvain, Louvain-la-Neuve, Belgium

**Keywords:** photodynamic therapy, immunogenic cell death, danger-associated molecular patterns (DAMPs), radiotherapy, vaccination

## Abstract

Recent studies have highlighted the potential of photodynamic therapy (PDT) to induce immunogenic cell death (ICD). The clinical use of photosensitizers (PS) to stimulate an anticancer immune response, and not to sterilize tumor cells, may however require some optimizations. Here, we examined how the dose of PS and the scheduling of PDT influence the generation of danger-associated molecular patterns proteins (DAMPs) and favor T cell antitumor activity. We found that upon photoactivation, a low dose of the non-porphyrinic PS OR141 was more prone than higher doses to induce DAMPs *in vitro* and to inhibit squamous cell carcinoma growth in mice. We further used PDT-killed cancer cells to prime dendritic cells (DC) and stimulate their maturation to evaluate whether the timing of their injection could influence the antitumor effects of radiotherapy. While PDT-based DC vaccination administered before radiotherapy failed to increase tumor growth inhibition, DC injection in the peri-radiotherapy period led to significant tumor growth delay, emphasizing the importance of the coincidence of T cell activation and alterations of the tumor bed. In conclusion, the use of OR141 as a bona fide ICD inducer led us to unravel both the non-linear relationship between PS concentration and PDT-induced antitumor immune response, and the value of an optimal timing of PDT when co-administered with conventional anticancer therapies. This study therefore stresses the necessity of adapting the clinical use of PDT when the goal is to promote an immune response and identifies PDT-based DC vaccination as a suitable modality to reach such objective.

## Introduction

Standard of care has evolved rapidly in the past years with the advent of immunotherapy as first line treatment for several malignancies including non-small cell lung cancer and melanoma (1). This paradigm shift originates from the impressive clinical impact of immune checkpoint inhibitors (ICI) that reinvigorate antitumor immune responses by interrupting co-inhibitory signaling pathways (2, 3). Today, a major challenge is to increase the types of cancer or for a given cancer, the patient subgroups who may benefit from immunotherapies. Besides the reduction in the immunosuppressive processes induced by cancer cells or promoted by the local tumor microenvironment (4–6), an increase in tumor immunogenicity may help to combat cancer immune evasion (7). The concept of immunogenic cell death (ICD) recently emerged as such a modality capable of activating tumor-targeted immune response through the emission of damage associated molecular patterns (DAMPs) (8). DAMPs result from the cellular redistribution and/or extracellular release of signals by dying cancer cells that experience endoplasmic reticulum (ER) stress (9–12). Currently, major treatments able to increase DAMPs and thereby to boost T cell response include oncolytic viruses (13), some chemotherapies like anthracyclines (14, 15) but also modalities such as radiotherapy (16, 17), high hydrostatic pressure (18), and photodynamic therapy with various photosensitizers (19, 20). These different ICD inducers actually share the capacity to exert strong pro-oxidant effects leading to protein misfolding which, at some stages, cannot be handled by the unfolded protein response (UPR) (12, 21). Recently, using a proprietary photosensitizer (PS), we have documented that proteasomal deubiquitinases (DUBs) USP14 and UCH37 were particularly sensitive to PDT-induced oxidation and that the resulting inhibition of their activities blocked proteasomal degradation of high MW oxidized protein aggregates, further increasing ER stress, and precipitating cell death (20).

Although the anticancer potential of ICD inducers is nowadays well-validated (8, 21, 22), there are still open questions related to the modality of their use in clinical settings. The dose and the time of administration are issues that are not straightforward. Indeed, while cell death needs to be induced to foster the release of some DAMPs, their upregulation and/or ectopic expression require a delay and thus potentially a submaximal dose to be optimal. This calls into question the use of maximal tolerated doses (MTD) that could kill cells too rapidly for the proper danger signaling pathway to occur. The same parameters may actually also influence the expression of MHC-I involved in tumor antigens presentation and safety issues may support the selection of a lower dose offering reasonable antitumor effects while minimizing toxicity in healthy organs. In the specific context of PDT that requires PS photoactivation to induce ER stress, the heterogeneity of tumor perfusion and the volume of the tumor mass *per se* may also alter the distribution of PS as well as the capacity of light to reach cancer cells in the depth of the tumor. While the latter issues may be circumvented by the *in vitro* PDT-based killing of cancer cells and further exposure to dendritic cells (DC), the timing of such DC-based vaccine administration may become an issue when combined with other anticancer modalities known to release tumor- associated antigens.

Here, we examined whether a proprietary photosensitizer OR141 (20, 23) may act as a *bona fide* ICD inducer and to which extent associated immune response is tunable according to the administered dose. Using DC exposed to PDT-killed cancer, we also investigated the importance of the PDT scheduling in particular when combined with radiotherapy.

## Materials and Methods

### Cell Culture and Treatments

Mouse SCC7 and human A431 squamous cell carcinoma cells as well as mouse B16 melanoma cells were initially acquired from collections where they are regularly authenticated by short tandem repeat profiling. Cells were used within 3 months after resuscitation from frozen aliquots and mycoplasma-free status was regularly confirmed. Cells were cultured in DMEM-Glutamax medium supplemented with 10% fetal bovine serum (FBS) and 1% penicillin/streptomycin 100x solution. For photodynamic therapy (PDT), cells were exposed to the benzophenazine photosensitizer OR141 (see Supplementary Figure 1) and illuminated with a 30 W equivalent day-light LED as previously reported [see (23) for absorption and output spectra, respectively]. Briefly, cells were washed and incubated in the dark for 1 h with OR141 at the indicated concentrations before washing with PBS and photoactivation with a day-light LED source (2.55 mW/cm^2^) for 1 h (9.18 J/cm^2^).

### Immunofluorescence

Cancer cells were seeded at low confluency in Nunc™-Lab-Tek™-II-Chamber-Slide™ (ThermoFischer) 24 h before staining. Cells were incubated with OR141 at the indicated concentrations for 30 min in the dark before incubation with ER-Tracker™ Red (ThermoFischer, ref. E34250) for 30 min. Nuclei were stained with Hoechst 33342 (Sigma, 2 μg/ml) for 30 min before mounting a coverslip with Dako Fluorescence mounting medium. Imaging was performed with AxioImager microscope (Zeiss) with 63X objective and fluorescence signal was analyzed with ImageJ software (24).

### Western Blot

For proteins extraction from supernatant (conditioned media), trichloroacetic acid (TCA) precipitation method was used. Briefly, cell culture medium was centrifuged to remove cell debris before incubation with 2% sodium deoxycholate (1/1,000 v/v) for 30 min on ice. TCA was then added to a final concentration of 7.5% and incubated on ice for 30 min. Proteins were recovered by high speed centrifugation (15,000 g for 20 min at 4°C) before two washing steps with ice-cold acetone and resuspension of the protein pellet in RIPA buffer. Immunoblotting was performed as previously described (20). Bip (Cell Signaling Tech., ref. 3177), Cleaved-PARP (Cell Signaling Tech., ref. 5625), Hsp90 (BD Biosciences, ref. 610419), Annexin A1 (Zymed, ref. 71–3,400), and HMGB1 (Abcam ref. ab18256) antibodies were diluted at 1/1,000 (v/v) and β-actin antibodies (Sigma, ref. A5441) at 1/2,500 (v/v) in a *TBST* (Tris-buffered saline, 0.1% *Tween* 20) solution with 1% w/v non-fat dry *milk*.

### Flow Cytometry

For cell death profiling, cells were treated with the indicated OR141 concentrations, trypsinized and washed with PBS. Cells were consecutively incubated with FITC-conjugated Annexin V (Immunostep, ANXVF-200T) and 1 μg/ml propidium iodide (PI, Sigma) according to manufacturer's instructions. After 15 min incubation at room temperature in the dark, cells were analyzed by flow cytometry on FACSCanto™ II (BD Biosciences) with a gating strategy excluding debris and doublet cells. For calreticulin translocation, cells were treated as described above and gently scrapped off the plate after 6 h. After staining with anti-calreticulin antibody (Abcam ref. ab22683), a secondary goat anti-mouse APC-coupled antibody was added for 15 min at RT. Cells were then counterstained with PI and analyzed on FACSCanto™II with a gating strategy excluding debris, doublet, and PI+ (dead) cells.

### HMGB1 and ATP Measurements

HMGB1 and ATP measurements were performed from conditioned media. Briefly, cells were grown to confluency in 6-well plates. Cells were treated with OR141 in low serum (1%) medium (without phenol red) and supernatants (SN) were recovered after 2 h (for ATP detection) or 24 h (for HMGB1 detection). After centrifugation to remove cell debris, SN were frozen until further analysis. ATP measurements were performed using a ENLITEN® ATP Assay (Promega, ref. FF2000) with the Glomax luminometer and HMGB1 amounts were measured using an ELISA kit (IBL International, ref. ST51011) according to the manufacturer's instructions.

### Mouse Experiments and Treatments

All the experiments involving mice received the approval of the University Ethic Committee (approval ID 2016/UCL/MD018), and were carried out according to National Animal Care regulations. C3H/HeNRj and NMRI Nude mice were obtained from Elevage Janvier (Le Genest-St-Isle, France) and C57bl/6j mice from Charles River (Saint-Germain-Nuelles, France). Tumor xenografts were initiated by injecting subcutaneously 2.5 × 10^5^ SCC7 cells in the flank of 7-week-old C3H or nude mice. Tumor volume was evaluated every other day with a digital caliper to determine width (w) and length (l) and by applying the following formula V=43∗π*(l/2)∗(w/2)2 (with l > w). Tumors were allowed to grow until 20 mm^3^ before initiating treatments. For PDT, OR141 was administered intraperitoneally (in Solutol/DMSO/NaCl 0.9%) and after 4 h, the tumor was illuminated for 1 h with a 30 W equivalent day-light LED as described above. For vaccination, 2 × 10^6^ DC (in 100 μl PBS) were injected subcutaneously three times at 1-week interval in the vicinity of the tumor draining lymph node (dLN); in the peri-radiation period, a fourth injection was occasionally used to prolong the response and evaluate possible tumor eradication. For irradiation, mice were anesthetised and placed on a lead deflector with a Ø10 mm hole centered on the tumor; irradiation was performed with an IBL Cesium-137 γ-ray irradiator.

### Dendritic Cells Culture and Vaccine Preparation

Dendritic cells were obtained from bone marrow of either 6-week-old C3H/HeNRj or C57bl/6j mice (Elevage Janvier and Charles River, respectively). Briefly, mice were euthanized and sprayed with 70% ethanol. Femur and tibia were removed and immersed for 5 min in 70% ethanol before washing in PBS. Both ends of bones were carefully sectioned and marrow was flushed using a 26G needle with culture medium (RPMI 1640 Glutamax medium supplemented with 10% FBS and 1% Penicillin/Streptomycin). Collected cells were centrifuged and exposed to a red blood cell lysis buffer (ThermoFischer, ref. 00-4333-57). Cells were washed 2 times before seeding (8 × 106 per 100-mm plate) in 10 ml DC medium (RPMI 1640 Glutamax supplemented with 10% FBS, 1% Pen/Strep, 200 μg/ml IL-4 (Peprotech, ref. 214–14 100 μg), 200 μg/ml GM-CSF (Peprotech, ref. 315–03 100 μg) and 50 μM β-mercaptoethanol. After 3 days, 4 ml of media were removed and replaced with 6 ml fresh medium. At day 7, the medium was again refreshed by replacing half of it (and recovering cells from the old medium by centrifugation). Bone marrow-derived dendritic cells (BM-DC) were either used at this stage (immature) or further matured by incubation with LPS (from *E. Coli* 0.5 μg/ml) or with cancer cells killed either by PDT (i.e., OR141 photoactivation) or three freeze-thawing cycles.

### CD8 Immunostaining

Tumors were surgically removed from mice at day 34 and fixed for 24 h in 4% PFA followed by overnight incubation in a 30% sucrose solution. Tumors were then snap-frozen in OCT and 5 μm-sections were obtained. For CD8 staining (Cell Signaling, ref. 98941), slides were fixed for 10 min in 4% PFA before antigen retrieval in citrate buffer (pH 5.7) with 0.5% Triton. Staining was performed following manufacturer guidelines with an AlexaFluor-488-conjugated secondary antibody (Invitrogen ref. A11034). Slides were scanned (3DHistech Pannoramic P250 Flash III, 20X) and the extent of CD8 staining was quantified over total tumor area using Visiopharm software.

### Statistics

Data are expressed as mean ± s.e.m. of at least three independent experiments. Statistical significance between experimental conditions was determined by Student's *t*-test or one-way analysis of variance (ANOVA, Tuckey's *post-hoc* test). All data were analyzed with GraphPad Prism 7.0 (San Diego, CA, USA).

## Results

### OR141 Non-linearly Influences ER Stress

**Figure 1:**
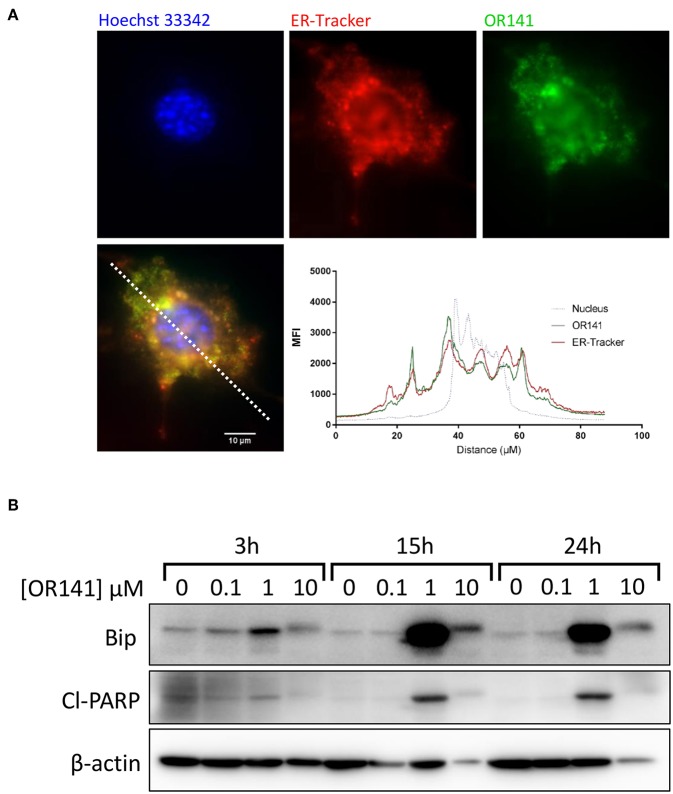
Non-linear induction of ER stress upon OR141 photoactivation. **(A)** Representative immunofluorescence pictures in squamous cell carcinoma SCC7 cells; nucleus (Hoechst 33342, blue), endoplasmic reticulum (ER-Tracker, red), and OR141 (auto-fluorescence, green); bottom panel shows superimposed fluorescence staining and corresponding signal intensity profile along the dotted white line. **(B)** Immunoblotting for Bip (GRP-78), cleaved-PARP (Cl-PARP), and β-actin (control) after the indicated incubation times in the presence of increasing concentrations of photoactivated OR141 in SCC7 carcinoma cells; note that beta-actin signals show heterogeneities due to recruitment in high MW aggregates in particular with the high PS concentrations. This experiment was repeated twice with similar results.

We previously demonstrated that photoactivation of OR141 leads to the generation of large amount of singlet oxygen (^1^O_2_), further inducing oxidation of endoplasmic reticulum (ER)-associated proteins and consecutive ER stress (20). Here, we first documented that OR141 (as detected by its green autofluorescence) co-localized perfectly with an ER-tracker (red fluorescence) in SCC7 squamous cell carcinoma cells giving rise to an overlapping intensity spectra (Figure 1A). We then examined the dose- and time-dependent changes, upon photoactivation obtained at constant light intensity and illumination duration (see section Materials and Methods), in the expression of the ER chaperone Bip as a reflection of the (UPR). The pattern of Bip upregulation was not strictly dose-dependent since the chaperone protein was more largely upregulated in the presence of 1 μM OR141 (than in response to the 10 μM dosage) (Figure 1B); this upregulation was detectable as soon as 3 h post-exposure to OR141. At the higher dose of 10 μM, Bip expression was only slightly higher than in control conditions. A small increase in the MW band detection was however consistently observed (Figure 1B), suggesting the formation of oxidized adducts involving Bip as previously reported for other proteins (20). Interestingly, the profile of Bip expression upon OR141 treatment correlated with the detection of cleaved-PARP as an apoptosis marker (Figure 1B).

### Cell Death Profile and Kinetics Vary According to the Photosensitizer Dose

**Figure 2:**
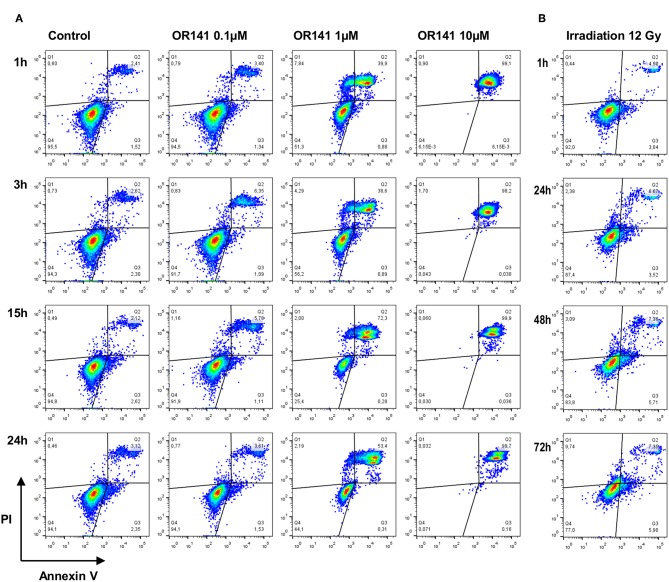
Differential cell death profile and kinetics following PDT and irradiation. Representative flow cytometry analysis of annexin-V and propidium iodide (PI) staining of squamous cell carcinoma SCC7 cells after the indicated incubation time and **(A)** in the presence of increasing concentrations of photoactivated OR141 and **(B)** upon exposure to ionizing radiations (12 Gy). This experiment was repeated twice with similar results.

To further explore the apparent discrepancies in OR141-induced ER stress according to the PS dose, we then used flow cytometry detection of annexin V (AnnV) and propidium iodide (PI) staining. While at the lowest dose of OR141 (0.1 μM) we did not observe significant cytotoxicity (vs. untreated conditions), SCC7 cell death was clearly detectable at the higher doses of 1 and 10 μM (Figure 2A). Differences between these two concentrations were however again detectable with a significant contribution of necrosis to cell death upon exposure to 1 μM, as detected by the increase in PI^high^ AnnV^low^ quadrant (Figure 2A). By contrast, in the presence of the highest OR141 concentration (i.e., 10 μM), SCC7 cells were readily found in the PI^high^ AnnV^high^ quadrant, as soon as 1 h after photoactivation, without transitioning by the PI^high^ AnnV^low^ quadrant (Figure 2A). In the presence of 1 μM OR141, late apoptosis (i.e., PI^high^ AnnV^high^) actually represented only 50% of the tested SCC7 cell population after 24 h (Figure 2A). Similar results were obtained with A431 squamous cell carcinoma cells; detection of cells in the PI^high^ AnnV^high^ quadrant was however slower than in SCC7 cancer cells and more A431 cells transitioned through the PI^low^ AnnV^high^ quadrant (Supplementary Figure 2A). As control experiments, we also evaluated the extent of SCC7 and A431 cancer cell death upon exposure to irradiation. Although using a high dose (12 Gy) and longer periods of incubation post-treatment, the number of dead cells remained marginal in comparison to PDT effects (Figure 2B and Supplementary Figure 2B).

### Generation of DAMPs and MHC-I Expression Are Induced at Low PS Dose

**Figure 3:**
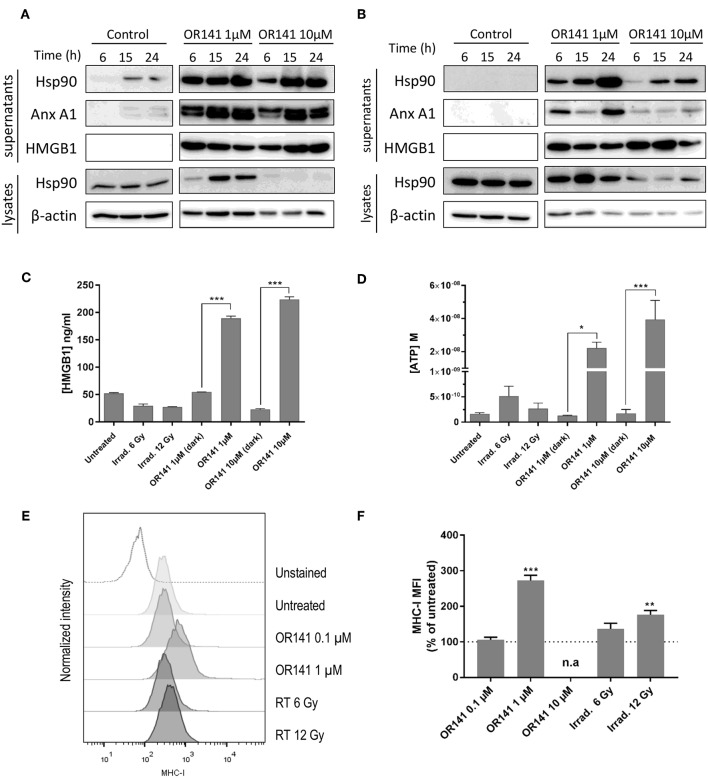
Low dose OR141 induces DAMPs and increases MHC-I expression. Representative immunoblotting for the indicated proteins either precipitated from the supernatants of squamous cell carcinoma SCC7 **(A)** and A431 **(B)** cells exposed to 1 or 10 μM photoactivated OR141 or from the corresponding cell lysates (*n* = 2–3); differences between doses was consistently observed in the lysates and after 6 h in the supernatants. **(C)** Immunoassay for HMGB1 released in the supernatant of SCC7 cells 24 h after the indicated treatments (*n* = 3). **(D)** Quantification of ATP released in the supernatant of SCC7 cells 2 h after the indicated treatments (*n* = 3). Representative flow cytometry histograms **(E)** and mean fluorescence intensity (MFI) **(F)** for MHC-class I molecule (H2Kk) expressed at the surface of SCC7 cells 48 h after exposure to increasing concentrations of photoactivated OR141 or ionizing radiations (*n* = 3); gating strategy excluded dead cells. n.a. = not applicable, *p < 0.05, **P < 0.01, ***P < 0.001.

We next studied the generation of danger-associated molecular patterns (DAMPs) following OR141 treatment. We found that Hsp90, Annexin A1, and HMGB1 were largely released in the extracellular medium in response to either 1 or 10 μM OR141 in SCC7 and A431 squamous cell carcinoma cells (Figures 3A,B). Interestingly, upon treatment with the lower OR141 concentration, a quicker increase in the extent of the above released DAMPs was observed (vs. 10 μM OR141, compare IB signals at 6 h in Figure 3A and at each time point in Figure 3B) together with an increase in the cytosolic abundance of Hsp90 (Figures 3A,B). Using other assays, we confirmed a significant increase in the extracellular release of HMGB1 and ATP regardless of the OR141 concentration (Figures 3C,D and Supplementary Figure 3A). Note however that the dramatic extent of cell death and associated loss of membrane integrity observed after 24 h exposure to 10 μM OR141 (see Figure 2 and Supplementary Figure 2) may have contributed to an unspecific release of cytosolic proteins; similar results were obtained with B16 cancer cells (Supplementary Figure 3B). The translocation of calreticulin to the plasma membrane, another well-known DAMP, could be detected by flow cytometry as early as 6 h post-PDT (Supplementary Figure 3C). However, since this measurement requires the use of living (non-permeabilized) cancer cells, it could not be performed at later stages with the high OR141 concentration (i.e., 10 μM), precluding comparison between low and high PS dosages. We finally used flow cytometry to document that PDT led to a significant increase in MHC-I expression in different cancer cells (Figures 3E,F and Supplementary Figure 3D). The optimal OR141 concentration to induce MHC-I was 1 μM since no effect was observed at 0.1 μM and the rapid cell death induction observed at 10 μM prevented such detection (Figure 3F). Of note, while radiotherapy failed to induce DAMPs (HMGB1 and ATP) in SCC7 cells (Figures 3C,D), a 12 Gy dose induced a slight increase in the expression of MHC class I molecules (Figures 3E,F); limited extent of DAMPs induced by radiotherapy may be related to the lesser cell death induced at the time of the assay (see Figure 2B).

### Anti-tumor Response of OR141 Is Non-linearly Dose-Dependent

**Figure 4:**
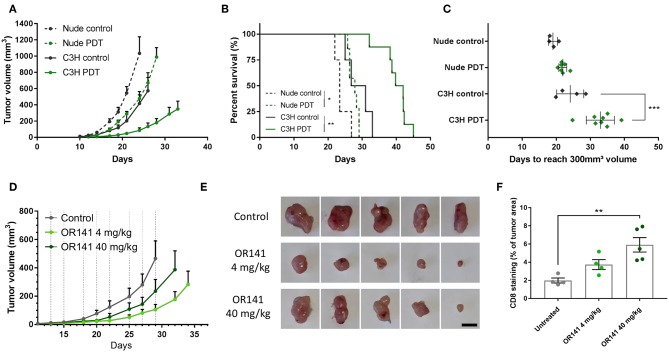
Low dose OR141 administration leads to a more pronounced inhibition of tumor growth than higher dose. Tumor growth curves **(A)**, survival **(B)** and calculated tumor growth delay **(C)** for nude and immunocompetent C3H mice injected s.c. with squamous cell carcinoma SCC7 cells and either left untreated (black, dotted and solid lines, respectively) or treated (green) 3-times a week with 40 mg/kg OR141 (i.p. injection followed 4 h later by light illumination of the tumor); *P < 0.05, **P < 0.01, ***P < 0.001, *n* = 5–8 mice per group. **(D)** Tumor growth curves for C3H mice injected s.c. with SCC7 cells and either left untreated (black) or treated with 4 and 40 mg/kg OR141 (light and dark green, respectively); *n* = 8 mice per group. **(E)** Pictures of representative tumors surgically removed from mice at day 34 (scale bar = 10 mm) **(F)**. Quantification of CD8+ T cells detected from whole tumor sections of tumors depicted in **(E)**; **P < 0.01, *n* = 4–5.

We then searched to verify that based on the above *in vitro* evidence, the *in vivo* antitumor response to OR141 was not strictly proportional to the PS concentration. Before comparing different doses of OR141, we aimed to validate the potential of an OR141-induced anticancer immune response (vs. direct cytotoxic effects) in our mouse models. For this purpose, we compared SCC7 tumor growth inhibitory effects in immunocompetent C3H/HeNRj and immunodeficient NMRI Nude mice. We observed that tumors grew more rapidly in nude mice than in immunocompetent mice (Figure 4A). More importantly, when exposed to OR141, a longer tumor growth delay was observed in immunocompetent mice (Figure 4A), leading to a significant increase in mouse survival (Figures 4B,C). In light of these results, we then compared the effects of different doses of OR141 on tumor growth in immunocompetent mice. Based on our previous work (20, 23), a dose of 40 mg/kg OR141 administered intraperitoneally leads to a peak serum concentration of 9.3 μM after 4 h, thus in the range of the higher dose tested *in vitro* (i.e., 10 μM). We therefore chose to compare the dose of 40 mg/kg with a 10-fold lower dose (4 mg/kg) and the highest achievable dose in terms of drug solubility (80 mg/kg). While no signs of toxicity were observed in the absence of illumination, upon photoactivation mouse treatment with this high dose had however to be prematurely stopped because skin toxicity (developing first as erythema) evolved toward non-scarring lesions; erythema was also observed in half of mice exposed to 40 mg/kg OR141 and light stimulation while the lowest dose did not induce any skin toxicity (Supplementary Figure 5A). Strikingly, despite this dose-dependency in the adverse effects, tumor growth was more largely inhibited by PDT with the 4 mg/kg dose than the 10-fold higher dose (Figures 4D,E). CD8 immunostaining however revealed a large infiltrate in tumors exposed to 40 mg/kg OR141 (Figure 4F and Supplementary Figure 5B), suggesting lymphocyte anergy in this condition. Note that the CD8 infiltrate tends to be lower in tumors exposed to the 4 mg/kg OR141 dose but this result is biased by the collection of smaller tumors that have objectively responded to PDT (see Figures 4E,F).

### Priming With PDT-Killed Cancer Cells Promotes Dendritic Cell Maturation

**Figure 5:**
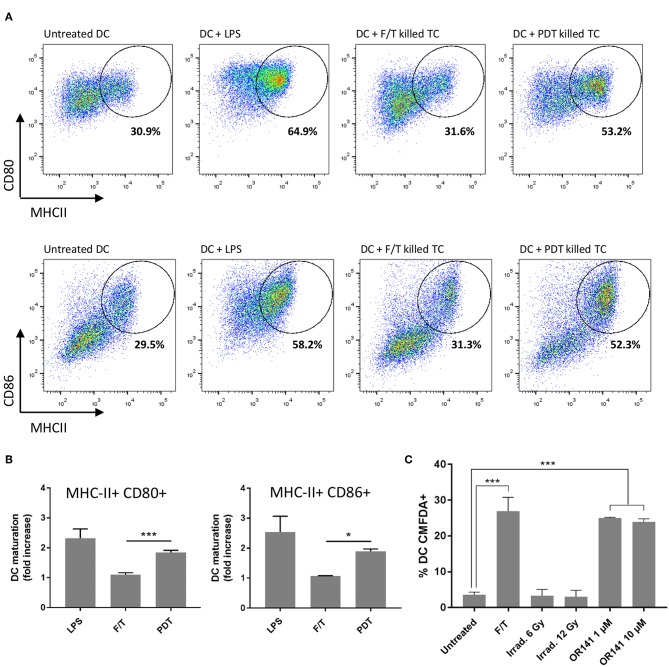
OR141-killed cancer cells promote DC maturation. **(A)** Representative flow cytometry analysis of cell-surface MHCII and either CD80 (top panels) or CD86 (bottom panels) staining of viable (eFluor 780-) CD11c+ bone marrow-derived DC (BM-DC) following incubation with LPS or SCC7 tumor cells (TC) killed either by freeze/thawing (F/T) or PDT (i.e., exposure to photoactivated OR141). **(B)** Bar graph depicts the corresponding changes in DC maturation based on the expression of the markers MHCII and either CD80 or CD86 (vs. untreated DC); *P < 0.05, ***P < 0.001, *n* = 3. **(C)** SCC7 cancer cells were stained with CMFDA, treated as indicated and incubated 24 h with BM-DC. CMFDA+ DC represent the proportion of DC that successfully phagocyte cancer cells; ***P < 0.001, *n* = 3.

After addressing the issue of PS dose, we aimed to examine to which extent the timing of PDT administration was critical when combined with other therapeutic modalities. We reasoned that the use of vaccination protocol based on the injection (at specific moments) of dendritic cells pre-challenged with *in vitro* PDT-killed cancer cells could facilitate the strict evaluation of temporal aspects. For this purpose, we first investigated whether PDT-killed cancer cells could promote the *in vitro* maturation of bone marrow-derived dendritic cells (BM-DC). We therefore evaluated the expression of maturation markers, namely MHC-II, CD80, and CD86, upon exposure to PDT-killed cancer cells. While freeze/thaw protocol for inducing cell death barely influenced the expression of either marker, PDT-killed cancer cells led to more than 50% MHC-II^+^ CD80^+^ CD86^+^ BM-DCs, almost reaching the numbers obtained with the TLR agonist LPS (Figures 5A,B); similar effects were obtained with another cancer cell line with >60% MHC-II^+^ CD80^+^ CD86^+^ BM-DCs (Supplementary Figure 6). It should be noted that the effects were independent of the process of antigen capture since CMFDA-prelabelled cancer cells killed either by freeze-thawing or PDT led to the same extent of phagocytosis (Figure 5C). Of note, the 10 μM OR141 dose did not lead to an increased extent of phagocytosis (vs. 1 μM OR141) and irradiated cancer cells failed to stimulate phagocytosis (Figure 5C).

### Timing of PDT-Based DC Vaccine and Radiotherapy Combination Influences Tumor Response

**Figure 6:**
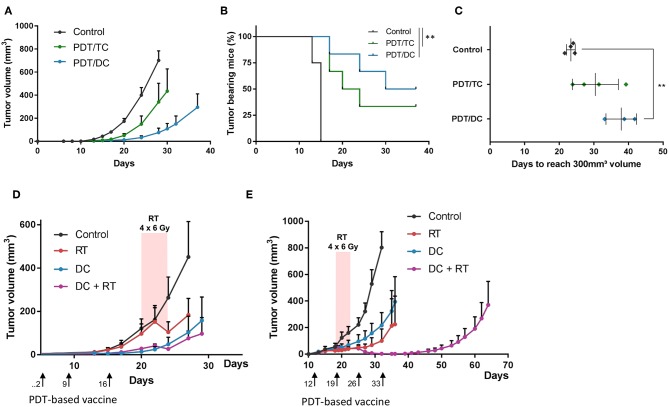
Administration of PDT-based DC vaccine reinforces the antitumor effects of radiotherapy when administered in the peri-radiation period. Tumor growth curves **(A)**, survival **(B)** and calculated tumor growth delay **(C)** for C3H mice injected s.c. with squamous cell carcinoma SCC7 cells and either left untreated (black lines) or treated 3-times a week with lysates of tumor cells (TC) killed by PDT (green) or dendritic cells (DC) pre-exposed to PDT-killed cancer cells (blue); **P < 0.01, *n* = 5–8 mice per group. **(D,E)** Tumor growth curves for C3H mice injected s.c. with SCC7 cells and either left untreated (black) or treated as indicated either with successive daily 6 Gy irradiation dose (red) or weekly administration of DC pre-exposed to PDT-killed cancer cells (blue) or both (purple). Vaccination either preceded radiotherapy **(D)** or was administered in the peri-radiation period **(E)**; *n* = 5–8 mice per group.

We next tested *in vivo* PDT-based vaccination protocols using primed DC either as monotherapy or in combination with radiotherapy. First, to validate the added value of using DC-based vaccine, we used mice bearing SCC7 tumors and treated them by administering either lysates of PDT-killed SCC7 cells or DC primed with PDT-killed SCC7 cells. In both tumor cell (TC) lysates- and DC-based vaccinated groups, tumor growth inhibition was observed, with however a longer growth delay in the group injected with DC (Figures 6A–C); mouse survival was observed in 40 and 50% of vaccinated mice with TC lysates and DC, respectively (Figure 6B). Of note, we also confirmed the migration of CMFDA-stained DC (after priming with PDT-killed cancer cells) in the draining lymph node located near the site of injection (Supplementary Figure 7A), leading to a net increase in the node size (vs. untreated conditions or exposure to non-primed DC) (Supplementary Figure 7B).

To determine whether the timing of PDT-based DC vaccination could influence the effects of radiotherapy, we irradiated SCC7 tumors when reaching 100 mm^3^ with four daily dose of 6 Gy and injected the DC vaccine according to two different schedules, either at day 2, 9, and 16 (i.e., before irradiation) (Figure 6D) or later on, when tumors were detectable by palpation at day 12 and then every other week (Figure 6E). While in the first protocol, DC vaccination did not increase the response obtained with irradiation, a significant increase in the tumor growth delay was observed with the second protocol when irradiation was performed in the peri-vaccination period with PDT-based DC (Figures 6D,E).

## Discussion

With the identification of PDT as an efficient modality to induce ICD, the use of PS is now increasingly considered as an adjuvant therapy for conventional anticancer modalities (19, 25–28). This renewed interest for PDT, used for decades as a cytotoxic approach (29–31), requires however to re-think the way it should be used. ICD indeed requires the release or the translocation of DAMPs (11) that may not necessarily be allowed to occur when rapid cell killing is the primary aim. In this study, we found that ICD induction is not linearly dose-dependent. A low PS dose actually induces a slower cell death with a maximal generation of DAMPs with similar and sometimes higher levels than those obtained with conventional PS MTD. This low dose effect was first identified *in vitro* and importantly recapitulated *in vivo* as the treatment of mice with 4 mg/kg OR141 led to more pronounced tumor growth inhibitory effects than when a 10-fold higher dose was used. By contrast, PDT-induced adverse effects such as erythema and non-scarring skin lesions in the vicinity of light-exposed area were dose-dependent and even required to discontinue treatment with the highest dose tested. Interestingly, the lesser antitumor effects obtained with the high PS dose could not be attributed to a deficit in lymphocyte recruitment since CD8^+^ cell infiltration was similar or even slightly larger than in response to the low PS dosage. This apparently counterintuitive result actually supports the multiple roles of PDT that besides DC maturation upon DAMPs release, stimulates immune cell infiltration upon induction of a local pro-inflammatory environment but also facilitates antigen presentation to promote T cell activity via MHC upregulation. While immune cell chemotaxis is proportional to the PS dose and exacerbated by other cytotoxic modalities (see below), our data indicate that increased tumor immunogenicity may require a submaximal PS dose to be optimal. It is worthy to note that the use of tumor cell lysates to prime DC has the potential to cover a broad spectrum of tumor-associated antigens (TAA) and thereby to maximize the chance to mount a complete immune response against tumor cell subpopulations. As in clinical trials exploiting this approach (32–35), we did not search for the identity of TAA implicated in the observed antitumor response. Our results obtained in a prophylactic vaccination assay (i.e., cell lysate injection followed 1 week later by mouse re-challenge with live tumor cells) however further support a TAA-driven memory immune response (Supplementary Figure 4).

Dendritic cells (DC)-based vaccination led us to explore another caveat related to the temporal aspects of the combination of PDT with another conventional anticancer modality, namely radiotherapy. Ionizing radiations have indeed the potential to cooperate with PDT-induced immune response by releasing tumor-associated antigens upon cancer cell killing and to create an environment favorable for the recruitment of primed immune cells (36, 37). When administered in tumor-bearing mice, we identified an additive effect of DC vaccination when delivered in the peri-radiation period. By contrast, no cooperation was observed when vaccination was administered before radiotherapy. The stimulatory effects of irradiation on the antitumor immune response may arise from a variety of mechanisms including the increased release and/or presentation of tumor antigens, the production of pro-inflammatory cytokines and the reversal of a suppressive tumor microenvironment via IFN-mediated signaling pathways (38–41). Radiotherapy effects are highly dose-dependent and while in our hands, the doses tested (i.e., 6 and 12 Gy) were not prone to induce DAMPs (at least as acutely as PDT), such high doses have been documented to be in the optimal window for immunostimulation through type I IFN production following dsDNA sensing by the cGAS/STING pathway (42, 43). In the current study, taking into account the required time to prime T cells upon DC administration, our data indicate that the above immunostimulatory effects of radiation on the tumor bed gain in being concomitant with the emergence of activated T cell populations and the maintenance thereof by consecutive DC injection. Another non-exclusive explanation is the killing or inactivation of tumor-infiltrated T cells when irradiation is delivered post-vaccination time. These data certainly emphasize that direct PDT delivery and more generally administration of ICD inducers require optimization to get the best of a combination with conventional anticancer therapy. Our findings actually reinforce the interest of using DC pre-challenged *in vitro* with PDT-killed cancer cells which offer the possibility to apply vaccination at the most appropriate time but also present several practical advantages. First, it allows an exact control of the dose delivered to cancer cells and thus an optimal ICD induction. Secondly, *in vitro* photoactivation gets rid of the constraints of light penetration in living tissues known to be dependent on the PS nature and the excitation wavelength (limited to a few centimeters at most). Finally, although toxicity of OR141 is by far more modest than classical porphyrins (20), the *ex vivo* use of PS considerably reduces the risk of toxicity associated with topical and systemic administration (44).

In conclusion, in this study, we validated OR141 PS as a promising ICD inducer and used it to document the need (i) to prefer a *low dose* of PS to maximize the immune response and (ii) to optimize the *timing* of PDT effects as revealed by vaccination with DC primed with PDT-killed cancer cells. These results open new perspectives for the use of PDT as an adjuvant modality to initiate/accentuate the immune response upon administration of conventional radio- or chemotherapy, but also as a particularly suited strategy to prime *in vitro* DC with killed cancer cells. For both options, PDT may thus provide personalized strategies by contributing to make antigens associated to a given tumor more prone to give rise to an anticancer immune response.

## Data Availability

The datasets generated for this study are available on request to the corresponding author.

## Author Contributions

BD, OR, and OF conceived the study, designed the experiments, and supervised the research. BD and OF wrote the manuscript. BD and NT performed the experiments. All the authors contributed to the interpretation of the results and critically revised the article.

### Conflict of Interest Statement

The authors declare that the research was conducted in the absence of any commercial or financial relationships that could be construed as a potential conflict of interest.
