# Supplementary material for: Low Photosensitizer Dose and Early Radiotherapy Enhance Antitumor Immune Response of Photodynamic Therapy-Based Dendritic Cell Vaccination
Source: Front Oncol. 2019 Aug 27;9:811. doi: 10.3389/fonc.2019.00811 (PMC6718637; doi:10.3389/fonc.2019.00811)
Supplement: Supplementary file 1 [file Data_Sheet_1.PDF]

Supplementary Material

Supplementary figure 1

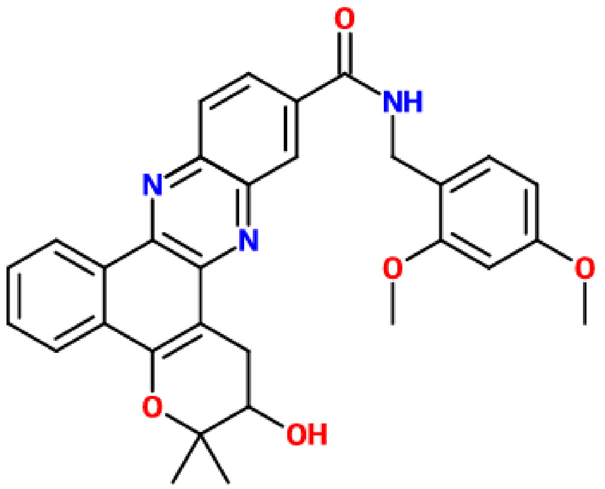

Supplementary figure 1. OR141 molecular structure.

Supplementary figure 2

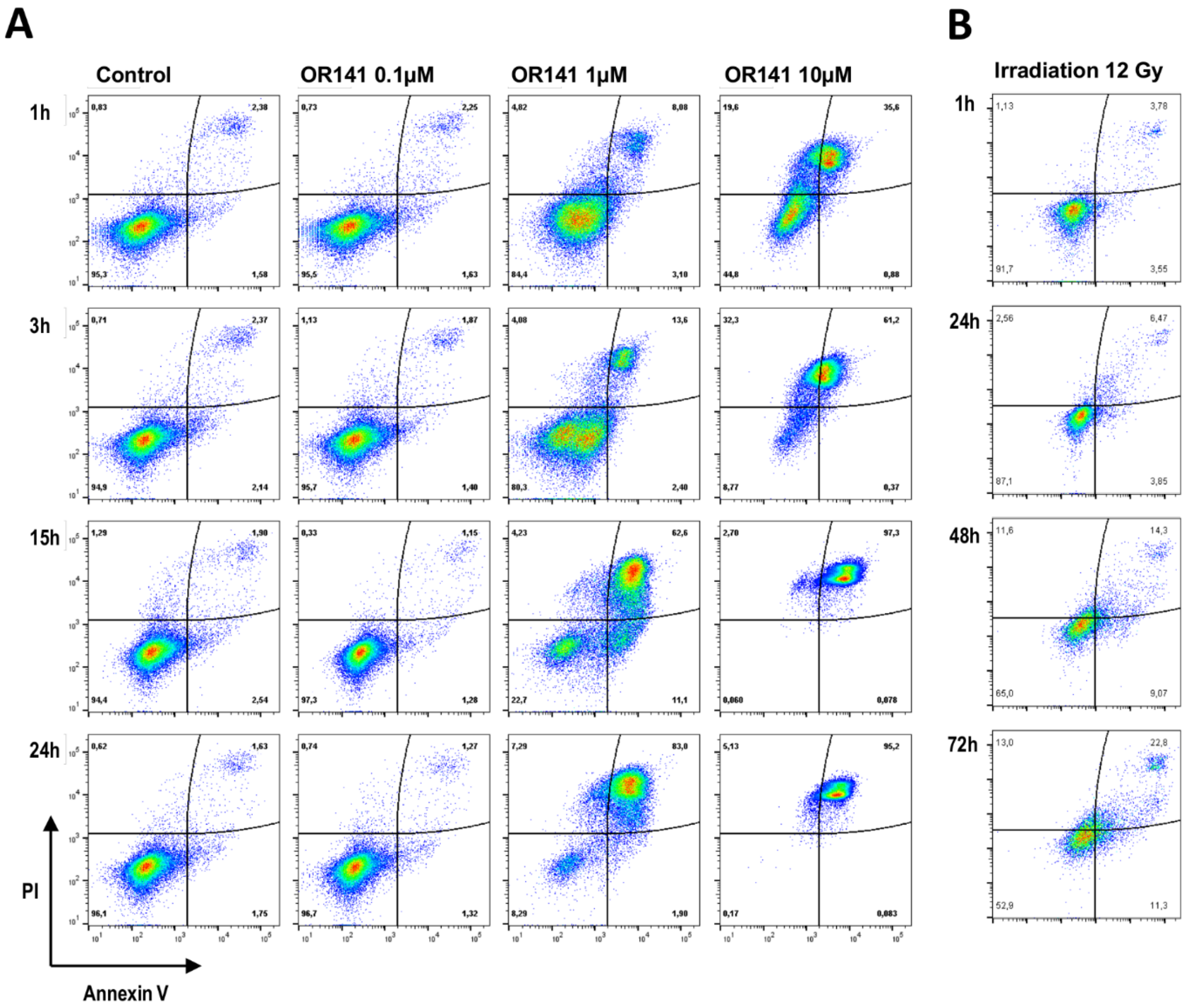

Supplementary figure 2. Differential cell death profile and kinetics following PDT and irradiation. Representative flow cytometry analysis of annexin-V and propidium iodide (PI) staining of squamous carcinoma A431 cells after the indicated incubation time and **(A)** in the presence of increasing concentrations of photoactivated OR141 and **(B)** upon exposure to ionizing radiations (12 Gy). This experiment was repeated twice with similar results.

Supplementary figure 3

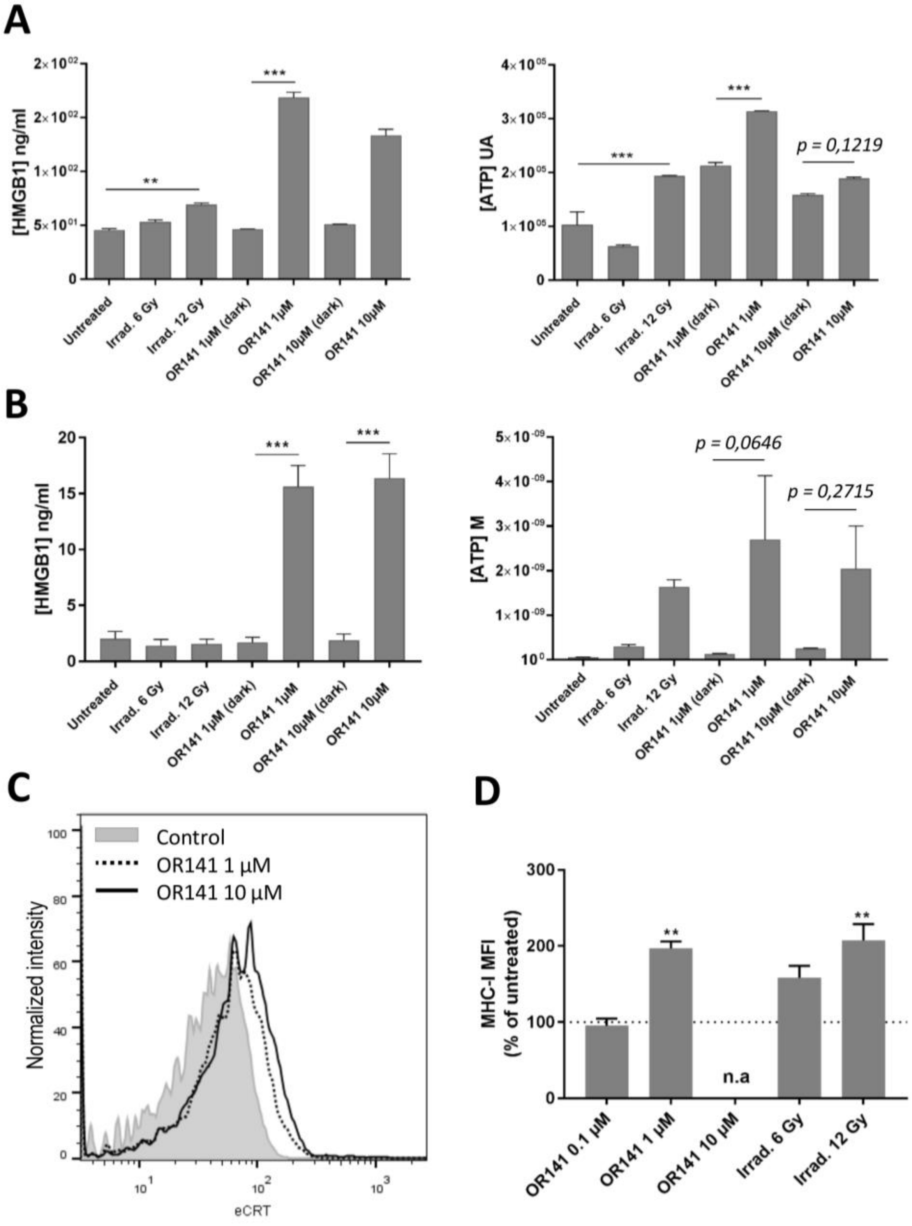

Supplementary figure 3. Low dose OR141 induces DAMPs and increases MHC-I expression. Immunoassay for HMGB1 (left) and quantification of ATP (right) released (after 24 hours and 2 hours, respectively) in the supernatant of A431 cells **(A)** and B16 melanoma cells **(B)** after the indicated treatments; **P<0.01, ***P<0.001, n=3. **(C)** Representative flow cytometry histogram for calreticulin translocation to the plasma membrane in squamous cell carcinoma SCC7 cells exposed for 6h to the indicated OR141 concentrations; gating strategy was used to only select living PI- cells (n=2). **(D)** Mean fluorescence intensity (MFI) for MHC-class I molecule expressed at the surface of B16 melanoma cells 48h after exposure to increasing concentrations of photoactivated OR141 or ionizing radiations; gating strategy excluded dead cells. n.a. = non applicable, **P<0.01 vs. untreated conditions, n=3.

Supplementary figure 4

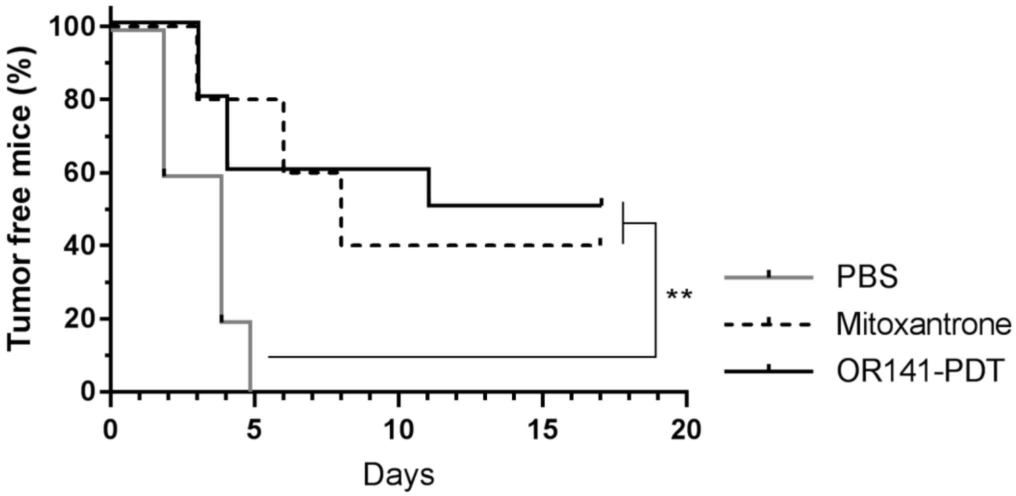

Supplementary figure 4. Prophylactic vaccination with OR141-killed cancer cells protects against subsequent challenge. Prophylactic vaccination of C3H mice with SCC7 cells killed by OR141-based PDT (black line), mitoxantrone (a known ICD inducer, dotted line) or negative control (PBS, grey line) before subsequent challenge with live cells in the opposite flank 7 days later. While prolonged survival was observed in 40-50 % mice vaccinated with OR141 and mitoxantrone-killed cancer cells, 100% mock vaccinated mice developed tumors ($P<0.01$, $n=10$).

Supplementary figure 5

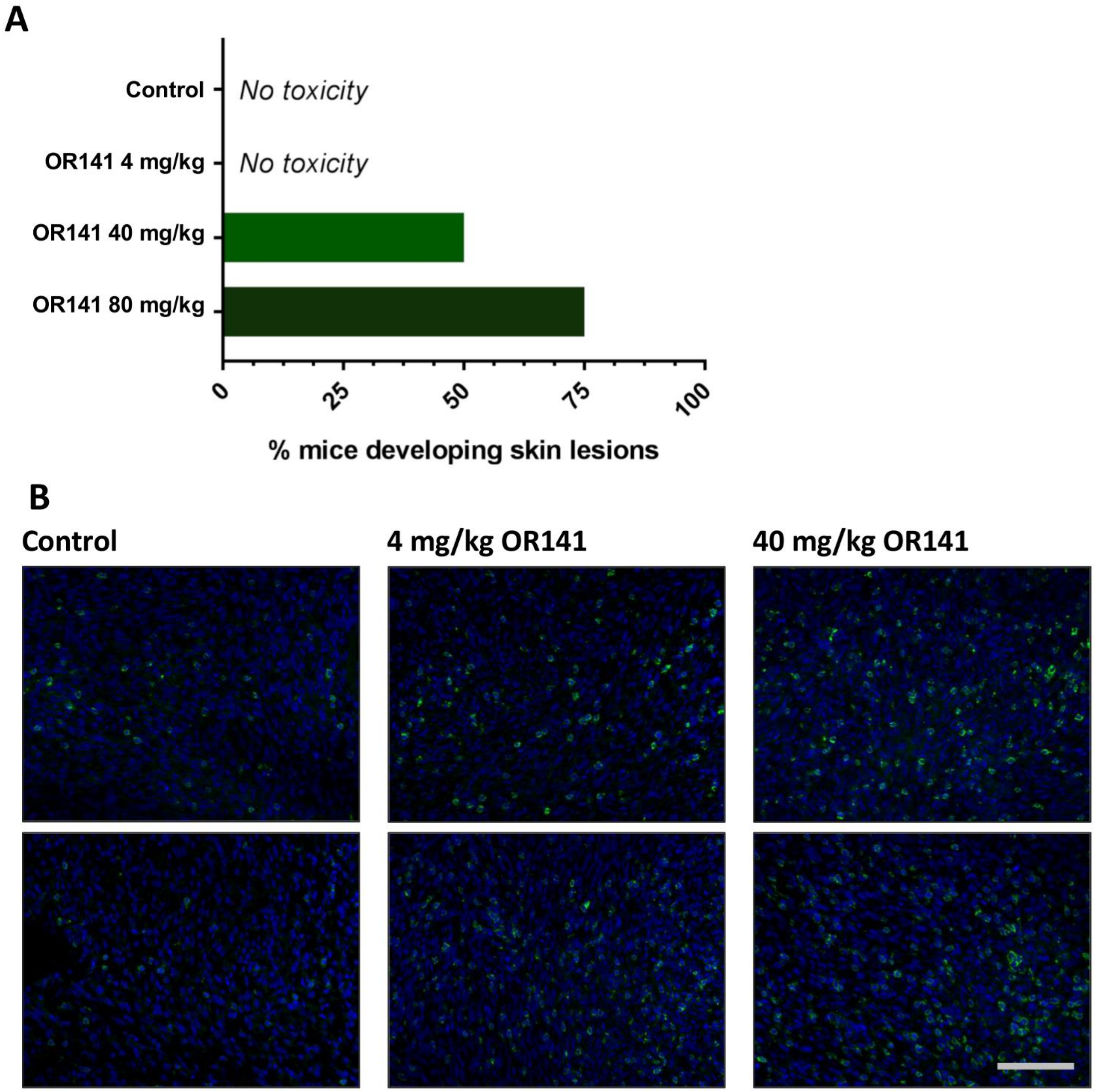

Supplementary figure 5. OR141 dose-dependent adverse effects and increase in tumor CD8 infiltration. (A) Graph depicting the extent of skin toxicity following mouse treatment with the indicated OR141 dose regimen (n=5-8); note that erythema evolve towards non-scarring skin lesions at the 80 mg/kg dose and necessitated early treatment discontinuation. **(B)** Representative pictures of CD8 staining (green) with DAPI nuclear staining (blue) on two tumor sections from either control mice or mice exposed to PDT with either 4 or 40 mg/kg OR141 (Scale bar: 100 µm).

Supplementary figure 6

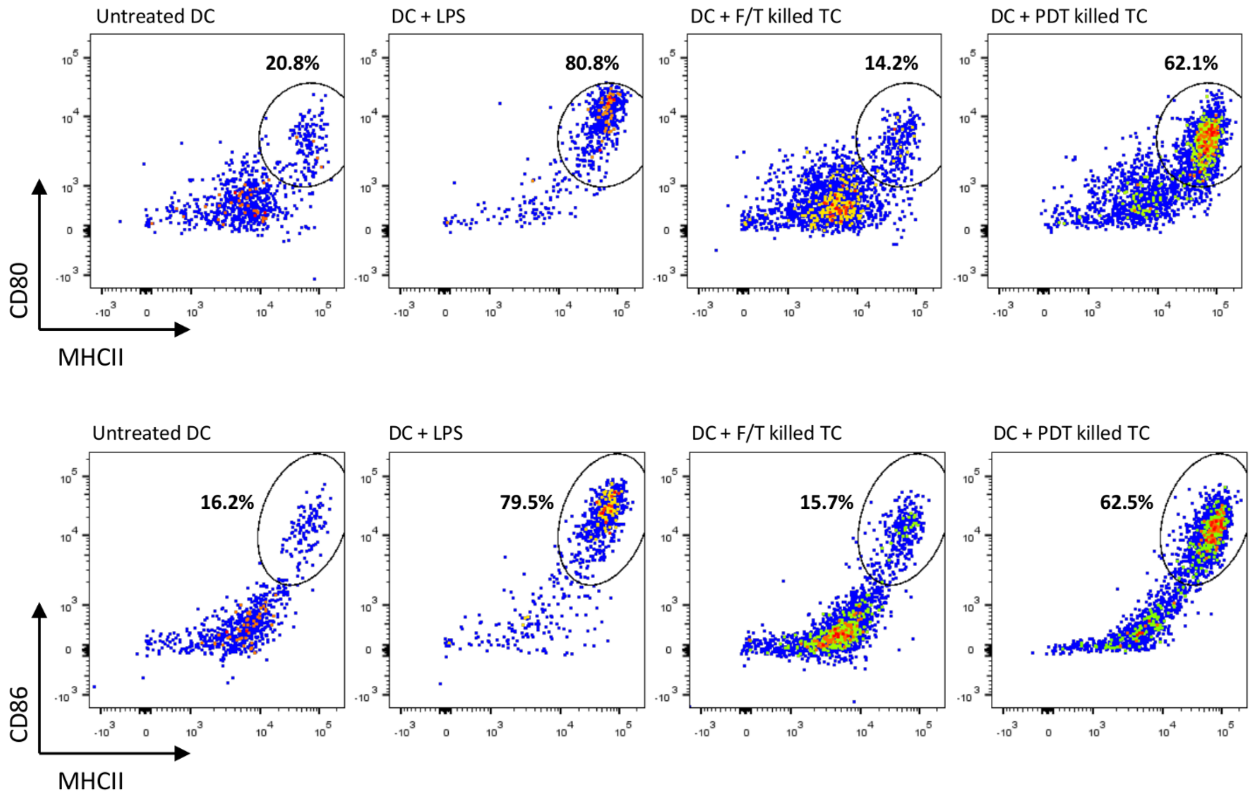

Supplementary figure 6. OR141-killed cancer cells promote DC maturation. Representative flow cytometry analysis of cell-surface MHCII and either CD80 (top panels) or CD86 (bottom panels) staining of viable (eFluor 780$^-$) CD11c$^+$ bone marrow-derived DC (BM-DC) following incubation with LPS or B16 tumor cells (TC) killed either by freeze/thawing (F/T) or PDT (i.e., exposure to photoactivated OR141). This experiment was performed twice with similar results.

Supplementary figure 7

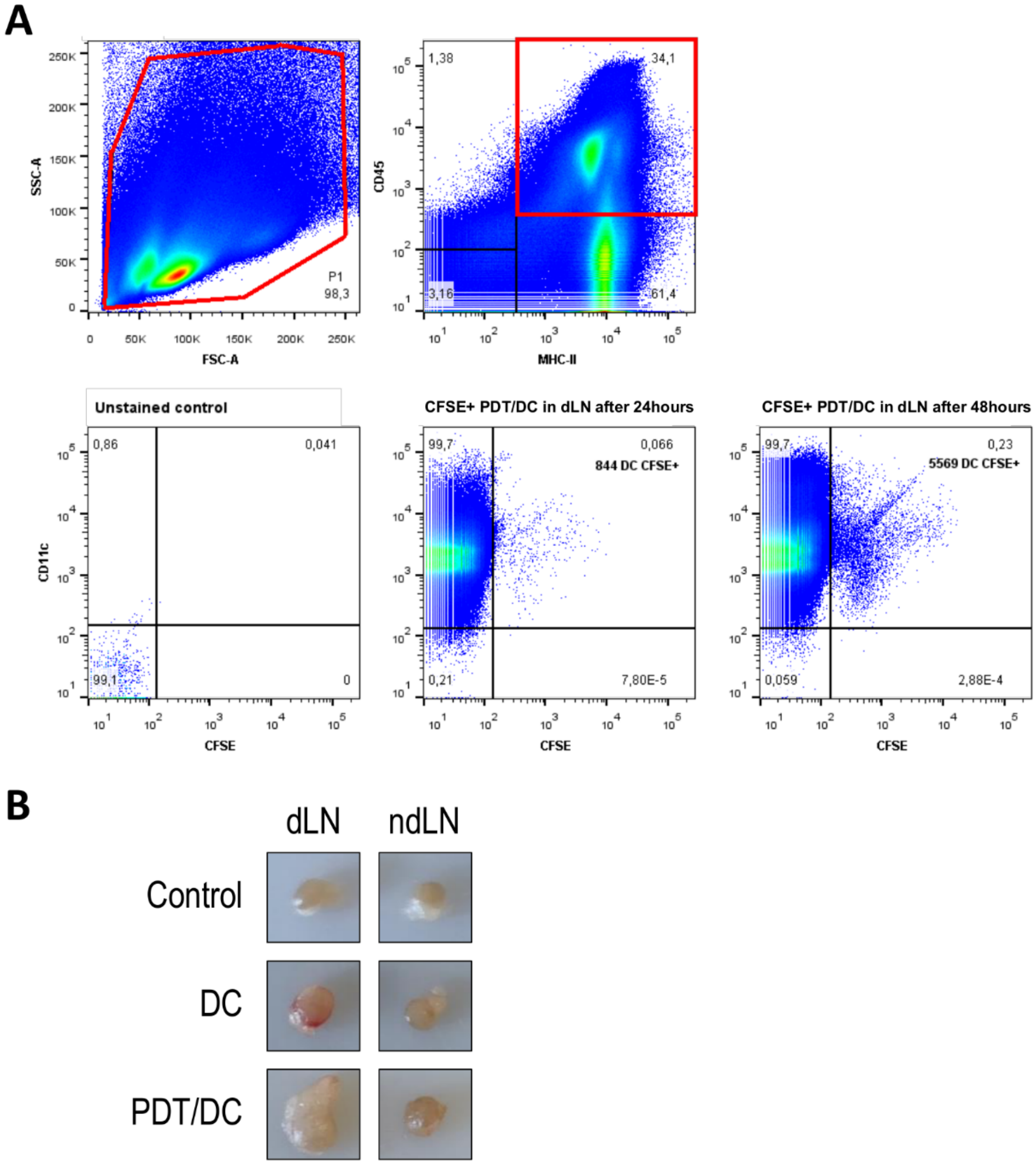

Supplementary figure 7. DC primed by PDT-killed cancer cells migrate to draining lymph node. (A) DC were stained with CFSE before injection in the lower quadrant of the mouse abdomen and the tumor-draining lymph nodes were collected after 24 and 48 hours. After dissociation, cells were analyzed by flow cytometry for the expression of CD45 and MHCII markers (top panels) and from this subpopulation, the amounts of $CD11c^{+}$ $CFSE^{+}$ cells were determined as depicted in the bottom panels. **(B)** Representative pictures of the inguinal draining lymph nodes (dLN) and non-draining lymph nodes (ndLN) from mice bearing SCC7 squamous cell carcinoma and either left untreated (control) or injected s.c. with either immature DC or DC primed with PDT-killed SCC7 cancer cells.
